# Associations among Heavy Metals and Proteinuria and Chronic Kidney Disease

**DOI:** 10.3390/diagnostics11020282

**Published:** 2021-02-11

**Authors:** Hui-Ju Tsai, Chih-Hsing Hung, Chih-Wen Wang, Hung-Pin Tu, Chiu-Hui Li, Chun-Chi Tsai, Wen-Yi Lin, Szu-Chia Chen, Chao-Hung Kuo

**Affiliations:** 1 Department of Family Medicine, Kaohsiung Municipal Ta-Tung Hospital, Kaohsiung Medical University Hospital, Kaohsiung Medical University, Kaohsiung 801, Taiwan; bankin_0920@yahoo.com.tw; 2 Research Center for Environmental Medicine, Kaohsiung Medical University, Kaohsiung 807, Taiwan; pedhung@gmail.com (C.-H.H.); 810064@kmuh.org.tw (W.-Y.L.); 3 Department of Pediatrics, Kaohsiung Medical University Hospital, Kaohsiung Medical University, Kaohsiung 807, Taiwan; 4 Department of Pediatrics, Kaohsiung Municipal Siaogang Hospital, Kaohsiung Medical University, Kaohsiung 812, Taiwan; 5 Department of Internal Medicine, Kaohsiung Municipal Siaogang Hospital, Kaohsiung Medical University, Kaohsiung 812, Taiwan; chinwin.wang@gmail.com (C.-W.W.); kjh88kmu@gmail.com (C.-H.K.); 6 Department of Internal Medicine, Division of Hepatobiliary, Kaohsiung Medical University Hospital, Kaohsiung Medical University, Kaohsiung 807, Taiwan; 7 Department of Public Health and Environmental Medicine, School of Medicine, College of Medicine, Kaohsiung Medical University, Kaohsiung 807, Taiwan; p915013@kmu.edu.tw; 8 Health Management and Occupational Safety Health Center, Kaohsiung Municipal Siaogang Hospital; Kaohsiung 812, Taiwan; 996117@kmsh.org.tw (C.-H.L.); 1036124@kmuh.org.tw (C.-C.T.); 9 Department of Internal Medicine, Division of Nephrology, Kaohsiung Medical University Hospital, Kaohsiung Medical University, Kaohsiung 807, Taiwan; 10 Department of Internal Medicine, Division of Gastroenterology, Kaohsiung Medical University Hospital, Kaohsiung Medical University, Kaohsiung 807, Taiwan

**Keywords:** heavy metals, proteinuria, chronic kidney disease, estimated glomerular filtration rate, renal function

## Abstract

Background: The prevalence of chronic kidney disease (CKD) is increasing annually in Taiwan. In addition to traditional risk factors, heavy metals contribute to the development of CKD. The aim of this study was to investigate associations among heavy metals and proteinuria and CKD in the general population in Southern Taiwan. We also explored the interaction and synergetic effects among heavy metals on proteinuria. Methods: We conducted a health survey in the general population living in Southern Taiwan between June 2016 and September 2018. Seven heavy metals were measured: blood lead (Pb) and urine nickel (Ni), chromium (Cr), manganese (Mn), arsenic (As), copper (Cu), and cadmium (Cd). Proteinuria was measured using reagent strips. CKD was defined as an estimated glomerular filtration rate (eGFR) of <60 mL/min/1.73 m^2^. Results: The mean age of the 2447 participants was 55.1 ± 13.2 years and included 977 males and 1470 females. Participants with high blood Pb and high urine Ni, Mn, Cu, and Cd were significantly associated with proteinuria. Interactions between blood Pb and urine Cr, and between urine Cd and Cu, had significant effects on proteinuria. The participants with high blood Pb and high urine Cu were significantly associated with an eGFR of <60 mL/min/1.73 m^2^. Conclusion: High blood Pb and high urine Cu may be associated with proteinuria and an eGFR of <60 mL/min/1.73 m^2^. High urine Ni, Mn, and Cd were significantly associated with proteinuria. Co-exposure to Cd and Cu, and Pb and Cr, may have synergistic effects on proteinuria.

## 1. Introduction

Environmental pollution such as heavy metals, air pollutants, agricultural chemicals, and contaminated drinking water and food is a major cause of disease, disability, and death worldwide [1], particularly as the global environment continues to worsen. Exposure to endocrine-disrupting or toxic chemicals plays an important role in disease initiation and progression [2], including respiratory diseases (such as asthma and chronic obstructive pulmonary disorder), neurobehavioral disorders (such as attention-deficit/hyperactivity disorder [3], depression, and other mental disorders), obesity and type 2 diabetes mellitus (DM) [4,5], and cancer [6]. Heavy metal exposure can cause various serious human diseases, such as respiratory problems, neurological disorders, and cancers [7]. Heavy metal exposure is also known to be a cause of acute and chronic kidney disease (CKD) [8], as are high-levels of occupational exposure [9]. The widespread use of heavy metals in industrial processes has resulted in environmental contamination of drinking water and soil, thereby increasing potential exposure among the general population [10]. However, the association between heavy metals and kidney problems in the general population remains poorly defined.

CKD is a major public health issue, and it can progress to end-stage renal disease (ESRD), with a reported prevalence ranging between 10.5% and 13.1% [11,12,13]. Patients with CKD have poor cardiovascular outcomes and a higher risk of mortality [14]. CKD is defined as evidence of kidney damage (such as albuminuria or proteinuria) and/or reduced kidney function (an estimated glomerular filtration rate (eGFR) of <60 mL/min/1.73 m^2^) for a period of at least three months [15]. Proteinuria is considered to be an early and essential diagnostic tool to evaluate disease severity and to monitor treatment response in several kidney diseases [16], and it is associated with an increased risk of ESRD and early death [17]. Taiwan has been reported to have the highest global incidence and prevalence rates of ESRD [18], and the annual incidence and prevalence rates increased by approximately three- and sevenfold, respectively, from 1990 to 2010 [18,19]. CKD is considered to be a multifactorial disease related to sex, age, obesity and smoking; chronic diseases such as metabolic diseases, DM, hyperlipidemia, hyperuricemia, hypertension and cardiovascular diseases; and also to genetic and environmental factors [7]. In addition to these tradition risk factors, numerous studies have suggested that heavy metals such as cadmium (Cd), lead (Pb), arsenic (As), mercury (Hg), uranium, and chromium (Cr) accumulate in the kidneys and that even low levels can induce CKD and proteinuria [9,20]. 

The aim of this study was to investigate the relationships among serum Pb, urine nickel (Ni), Cr, manganese (Mn), As, copper (Cu), and Cd and proteinuria and CKD in the general population in Southern Taiwan. We also explored the interactions and synergetic effects among these heavy metals on proteinuria. 

## 2. Materials and Methods

### 2.1. Subject Recruitment 

We conducted a health survey of the general population living in Southern Taiwan from June 2016 to September 2018. Participants were recruited through advertisements, and those who were willing to attend the study were included. All participants had face-to-face interviews during which anthropometric variables (weight and height) were measured, physical examinations were performed, and medical histories were recorded by an experienced physician. 

### 2.2. Collection of Demographic, Medical, and Laboratory Data 

Baseline variables were recorded, including systolic blood pressure (SBP) and diastolic blood pressure (DBP); laboratory data (fasting glucose, triglycerides, total cholesterol, high-density lipoprotein (HDL)-cholesterol, low-density lipoprotein (LDL)-cholesterol, hemoglobin, eGFR, and uric acid); medical history (DM and hypertension); occupation history; living environment; and demographic characteristics (age and sex). The eGFR was calculated using the Chronic Kidney Disease Epidemiology Collaboration (CKD-EPI) equation [21]. Body mass index (BMI) was calculated as weight in kilograms divided by height in meters squared.

### 2.3. Measurement of Blood and Urine Heavy Metals 

A total of seven heavy metals were measured: Pb in the blood, and Ni, Cr, Mn, As, Cu, and Cd in the urine. Concentrations of blood Pb (AA800v, PerkinElmer) and the other heavy metals in urine (ICP-MS, NexION 300 Series, Perkin Elmer) were analyzed using graphite furnace atomic absorption spectrometry as described in the National Institute of Environmental Research.

### 2.4. Definition of Proteinuria and CKD

Proteinuria was measured using reagent strips (Hema-Combistix, Bayer Diagnostics). A test result of 1+ or higher was defined as being positive. CKD was defined as an eGFR of <60 mL/min/1.73 m^2^ depending on the KDOQK/DOQI clinical practice guidelines [22].

### 2.5. Ethics Statement 

The Institutional Review Board of Kaohsiung Medical University Hospital approved the study protocol (number: KMUHIRB-G(II)-20190011). All participants provided informed consent before participating in the study.

### 2.6. Statistical Analysis

Data are presented as percentages, means ± standard deviations (SD), or medians (25th–75th percentile) for heavy metals and triglycerides. The chi-square test was used to test between-group differences for categorical variables, and the independent t-test was used for continuous variables. Multivariable logistic regression analysis was used to examine associations among the heavy metals and proteinuria and CKD. Interactions among heavy metals and their effects on proteinuria were analyzed using logistic regression analysis. The interaction effects of the heavy metals on proteinuria were illustrated using the SGPLOT procedure. Multivariable linear regression analysis was used to identify associations between heavy metals and eGFR. Receiver operating characteristic (ROC) curves and areas under the curves (AUCs) were used to assess the performance and predictive abilities, respectively, of the heavy metals in identifying proteinuria and an eGFR of <60 mL/min/1.73 m^2^. For all heavy metals (in both blood and urine), natural logarithms were used. A *p* value of less than 0.05 was considered to indicate a statistically significant difference. All statistical analyses were conducted using SPSS version 19.0 for Windows (SPSS Inc. Chicago, USA). 

## 3. Results

The mean age of the 2447 participants was 55.1 ± 13.2 years and included 977 males and 1470 females. The overall prevalence rates of proteinuria and eGFR <60 mL/min/1.73 m^2^ were 10.3% and 6.3%, respectively. A comparison of the clinical characteristics between the participants with and without proteinuria is shown in Table 1. Compared to the participants without proteinuria, those with proteinuria were older, more frequently male, and more likely to be jobless; had higher prevalence rates of DM and hypertension; and had higher values of BMI, SBP, DBP, fasting glucose, triglycerides, and uric acid. However, those without proteinuria had lower values of total cholesterol, HDL-cholesterol, LDL-cholesterol, and eGFR, and they were less likely to decorate their house in the past six months. Regarding heavy metals, the participants with proteinuria had higher levels of blood Pb and urine Ni, Mn, Cu, and Cd. 

### 3.1. Determinants of Proteinuria

Table 2 shows the determinants of proteinuria in the study participants. After adjusting for each heavy metal and for age, sex, DM, hypertension, BMI, DBP, SBP, occupation, house decoration in the past six months, log triglycerides, fasting glucose, total cholesterol, HDL-cholesterol, LDL-cholesterol, eGFR, and uric acid (significant variables in Table 1), the participants with high blood Pb (log per 1 μg/dL; odds ratio (OR), 3.089; 95% confidence interval (CI), 1.630 to 5.853; *p* = 0.001), high urine Ni (log per 1 μg/L; OR, 3.642; 95% CI, 2.285 to 5.807; *p* < 0.001), high urine Mn (log per 1 μg/L; OR, 2.443; 95% CI, 1.649 to 3.619; *p* < 0.001), high urine Cu (log per 0.1 μg/dL; OR, 1.945; 95% CI, 1.750 to 2.162; *p* < 0.001), and high urine Cd (log per 1 μg/L; OR, 2.671; 95% CI, 1.733 to 4.118; *p* < 0.001) were significantly associated with proteinuria. However, urine Cr and As were not significantly associated with proteinuria.

**Figure 1:**
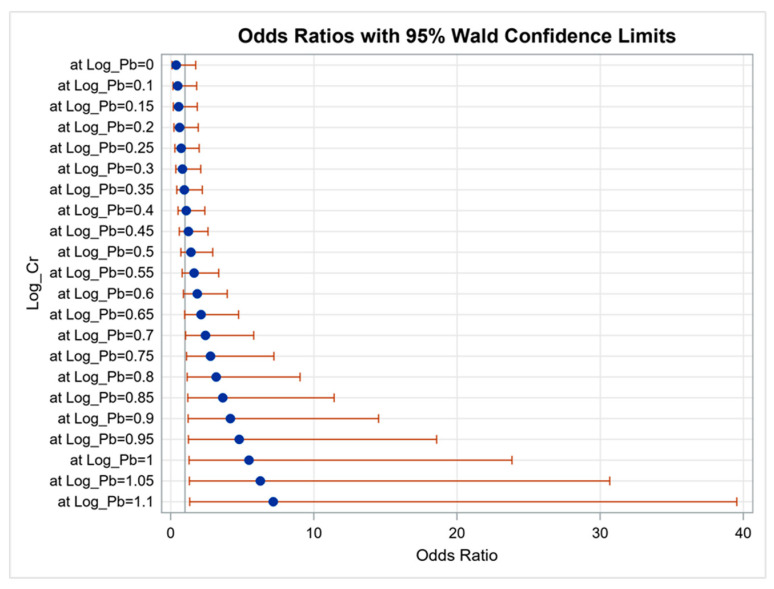
Synergistic effect of blood Pb and urine Cr on proteinuria. The interaction between blood Pb and urine Cr on proteinuria was statistically significant (*p* = 0.047).

**Figure 2:**
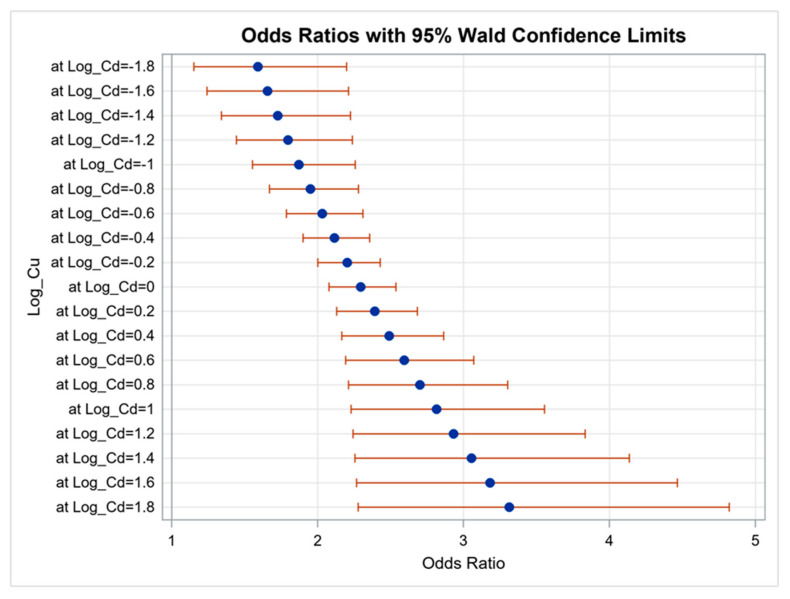
Synergistic effect of urine Cd and Cu on proteinuria. The interaction between urine Cd and Cu on proteinuria was statistically significant (*p* = 0.033).

We analyzed the effects of interactions between the heavy metals on proteinuria using logistic regression analysis. The results showed that the effects of interactions between blood Pb and urine Cr (OR, 14.846; 95% CI, 1.032 to 213.663; *p* = 0.047) and urine Cd and Cu (OR, 1.226; 95% CI, 1.017 to 1.478; *p* = 0.033) on proteinuria were statistically significant. However, interactions of other combinations did not achieve significance. Figure 1 illustrates the synergistic effect of blood Pb and urine Cr on proteinuria. When log Pb = 0.70, every additional unit of log Cr increased the risk of proteinuria by 2.43 times (*p* = 0.0460, Supplement Table S1). Figure 2 illustrates the synergistic effect of urine Cd and Cu on proteinuria. When log Cd = 0.0, every additional unit of log Cr increased the risk of proteinuria by 2.30 times (*p* < 0.001, Supplement Table S2).

### 3.2. Determinants of CKD

Table 3 shows a comparison of the clinical characteristics among the participants with and without an eGFR of <60 mL/min/1.73 m^2^. Compared to the participants without an eGFR of <60 mL/min/1.73 m^2^, those with an eGFR of <60 mL/min/1.73 m^2^ were older, more frequently female, more likely to be jobless, and more likely to burn incense; had higher prevalence rates of DM and hypertension; and had higher values of SBP, fasting glucose, triglycerides, and uric acid. However, those with an eGFR of <60 mL/min/1.73 m^2^ had lower values of hemoglobin and were less likely to decorate their house in the past six months. Regarding heavy metals, the participants with an eGFR of <60 mL/min/1.73 m^2^ had higher levels of blood Pb and urine Ni, As, and Cu.

Table 4 shows the determinants of an eGFR of <60 mL/min/1.73 m^2^ in the study participants. After adjusting for each heavy metal and for age, sex, DM, hypertension, SBP, occupation, house decoration in the past six months, burned incense, fasting glucose, log triglycerides, hemoglobin, and uric acid (significant variables in Table 3), the participants with high blood Pb (log per 1 μg/dL; OR, 3.727; 95% CI, 1.207 to 11.510; *p* = 0.022) and high urine Cu (log per 0.1 μg/dL; OR, 1.163; 95% CI, 1.038 to 1.303; *p* = 0.009) were significantly associated with an eGFR of <60 mL/min/1.73 m^2^. However, urine Ni, Cr, Mn, As, and Cd were not significantly associated with an eGFR of <60 mL/min/1.73 m^2^.

### 3.3. ROC Curve Analysis for Heavy Metals in Identifying Proteinuria and eGFR < 60 mL/min/1.73 m^2^

**Figure 3:**
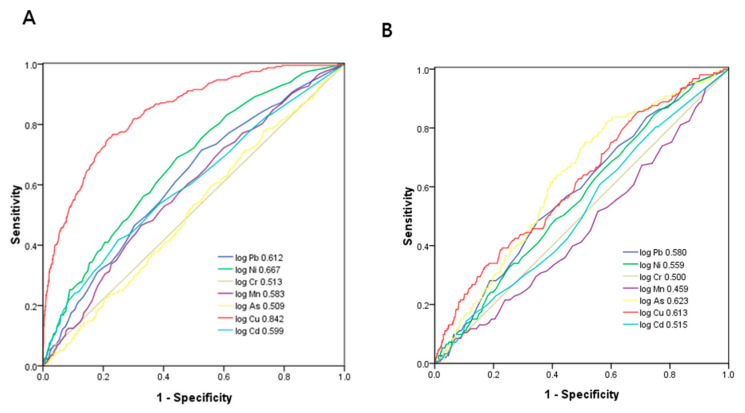
Comparison of the AUCs of seven heavy metals for identifying (**A**) proteinuria and (**B**) eGFR < 60 mL/min/1.73 m^2^.

Figure 3a demonstrates the ROC analysis and AUCs of seven heavy metals in identifying proteinuria. Among these heavy metals, Cu had the greatest AUC (AUC = 0.842), followed by Ni (AUC = 0.667), Pb (AUC = 0.612), Cd (AUC = 0.599), Mn (AUC = 0.583), Cr (AUC = 0.513), and As (AUC = 0.509). Table 5 demonstrates the ROC analysis and AUCs, the cutoff values, the Youden index values, and the sensitivity and specificity of seven heavy metals for proteinuria.

Figure 3b demonstrates the ROC analysis and AUCs of seven heavy metals in identifying eGFR < 60 mL/min/1.73 m^2^. Among these heavy metals, As had the greatest AUC (AUC = 0.623), followed by Cu (AUC = 0.63), Pb (AUC = 0.580), Ni (AUC = 0.559), Cd (AUC = 0.515), Cr (AUC = 0.500), and Mn (AUC = 0.459). Table 6 demonstrates the ROC analysis and AUCs, the cutoff values, the Youden index values, and the sensitivity and specificity of seven heavy metals for eGFR <60 mL/min/1.73 m^2^.

## 4. Discussion

In this study, we found that the participants with high levels of blood Pb and urine Cu may be associated with proteinuria and an eGFR of <60 mL/min/1.73 m^2^. In addition, the participants with high levels of urine Ni, Mn, and Cd were significantly associated with proteinuria. A synergistic effect of urine Cd and Cu on proteinuria was also observed. Although urine Cr was not significantly related to proteinuria, a synergistic effect of blood Pb and urine Cr on proteinuria was observed. 

The first important finding of this study is that high blood Pb may be associated with proteinuria and an eGFR of <60 mL/min/1.73 m^2^. In ROC curve analysis among seven heavy metals, blood Pb had the third highest predictive performance to identify proteinuria and an eGFR of <60 mL/min/1.73 m^2^. Factors known to be associated with high Pb levels include the male sex, older age, low socioeconomic status, smoking, living in urban areas and older buildings, and Pb in paint and water pipes [23]. Pb accumulates in and is excreted by the kidneys [24], and there is convincing evidence to support a direct relationship between Pb exposure and several kidney diseases [8,25,26]. Pb exposure can cause oxidative stress in tubular and glomerular cells, leading to the generation of free radicals, which can contribute to cellular apoptosis and subsequent changes in renal structure and function [27]. Acute Pb intoxication can disturb solute and amino acid transport in renal tubules, leading to proximal tubular dysfunction, such as Fanconi syndrome [8,26]. In addition, chronic intoxication can cause progressive tubulointerstitial nephritis, glomerular sclerosis, and tubular atrophy [25]. Epidemiologic studies have shown a positive association between chronic low Pb exposure (blood Pb levels of <5–10 mg/dL) and reduced renal function [23,28]. Our findings suggest that the participants with high blood Pb may be associated with proteinuria and an eGFR of <60 mL/min/1.73 m^2^, which is consistent with previous studies. 

The second important finding of this study is that high urine Cu was associated with proteinuria and an eGFR of <60 mL/min/1.73 m^2^. In ROC curve analysis among seven heavy metals, urine Cu had the highest predictive performance to identify proteinuria and the second highest predictive performance to identify an eGFR of <60 mL/min/1.73 m^2^. Cu is the third most abundant essential transition metal in humans, and it is a cofactor of several enzymes involved in a number of physiological pathways. Humans are primarily exposed to Cu through food intake, and it reaches the kidneys via circulation [20]. In the kidneys, Cu catalyzes the generation of highly reactive hydroxyl radicals, and this oxidative stress can cause proximal tubule necrosis [29,30]. Few epidemiological studies have investigated the potential relationship between Cu exposure and renal function. Yang et al. conducted a population-based cross-sectional study in China and found that urine Cu (>20.92 μg/L) was associated with an abnormal eGFR (an eGFR of <60 mL/min/1.73 m^2^) [31]. Another study enrolled 194 CKD patients in Taiwan and reported a significantly increasing trend of serum Cu with advanced stages of CKD [32]. This study also proposed that high urine Cu may increase the risk of proteinuria and a reduced eGFR. 

Previous studies have discussed the relationship between Mn and CKD, however the results have been inconsistent [33,34]. A cross-sectional study in Spain found that predialysis patients with CKD were associated with higher circulating levels of Mn than controls [33]. However, Liu et al. conducted a prospective study to evaluate the associations between plasma metal levels and a decline in kidney function among middle-aged and elderly Chinese and did not find an association between plasma Mn and reduced renal function [34]. Another study from China reported that plasma Mn was negatively associated with CKD in people aged ≥90 years [35]. In the present study, we found that high urine Ni and Mn levels were associated with proteinuria. Nickel mainly accumulates in and is excreted by the kidneys [20]. Excess Ni has been shown to trigger an inflammatory response by activating nuclear factor-κB and tubular apoptosis through the phosphoinositide 3-kinase (PI3K)–RAC serine/threonine-protein kinase (AKT) pathway [20,36]. A retrospective study in Changhua, Taiwan, found that high urine Ni was a risk factor for ESRD progression (log per 1 mg/kg, adjusted hazard ratio = 1.08, CI = 1.00–1.17) [37]. 

Another important finding of this study is that high urine Cd was associated with proteinuria and that it had a synergistic effect with urine Cd and Cu on proteinuria. Cd is known to be a nephrotoxic environmental pollutant [10]. High levels of exposure can result in the accumulation of Cd in the proximal tubules of the kidney, and this has been shown to impair tubular function and protein reabsorption [38]. The most common long-term adverse effect of exposure to Cd is proteinuria [26]. Increasing evidence has shown that chronic exposure to Cd is associated with a reduced eGFR and an increased risk of CKD [20,26]. In the present study, the participants with high urine Cd had a higher risk of proteinuria. Exposure to more than one heavy metal is common, and combined exposure may enhance nephrotoxicity. We observed the synergistic effect of urine Cd and Cu on the association with proteinuria in this study. Cd has been shown to interact metabolically with metals including Cu [39]. The ions of these metals are competitively transported and tightly bound to metallothionein in the systemic circulation [40,41]. Therefore, the concentration of other essential metals can be affected by the concentration of Cd in the human body. Eom et al. hypothesized that Cu imbalance may be a determinant of oxidative stress and renal tubular damage in people with chronic exposure to Cd. Further studies are needed to explore the synergistic effects of co-exposure to metals with regards to nephrotoxicity. 

Epidemiological studies have reported a link between occupational Cr exposure and kidney damage [42,43]. A study of 360 Taiwanese adults aged 19–84 years from the National Nutrition and Health Survey in Taiwan from 2005 to 2008 reported a significant association between Cr exposure and reduced renal function [44]. Co-exposure to Cr with Pb and Cd was also potentially associated with an additional decline in eGFR [44]. In the present study, we did not find a significant association between urine Cr and proteinuria after adjustments. However, we did find a synergistic effect of blood Pb and urine Cr on proteinuria. The mechanism of Cr-associated nephrotoxicity is unknown. The kidney is considered to be a critical organ for Cr toxicity, and trivalent and hexavalent Cr compounds have been reported to accumulate in proximal convoluted tubules [45]. Cr-induced cytotoxicity, DNA damage, and oxidative stress have also been reported in animal kidneys [46,47]. This possible interactive toxic effect of Cr and Pb may be explained by their synergistic effect on oxidative stress via the mitogen-activated protein kinase pathway [48,49].

There are several limitations to this study. First, the long-term clinical outcomes and causal relationships could not be confirmed given the cross-sectional study design. Long-term prospective studies with serial measurements of heavy metals and kidney function are required to verify our results. Second, proteinuria was measured using a reagent strip, not by quantitative estimation of proteinuria such as a spot urine protein-creatinine ratio or a 24-h urine collection. Nevertheless, urine reagent strips have been shown to have high sensitivity and a specificity of >90%, when using a urine albumin-to-creatinine ratio of ≥300 mg/g as the reference standard [50]. Third, the use of a single measurement to define metal exposure is an important limitation. In addition, renal dysfunction causes less clearance of heavy metals, resulting in a high serum concentration of the materials, which may produce an incorrect interpretation. Fourth, total urine was used to reflect the toxic form of inorganic As exposure. Total urine As can be measured quickly for a large number of samples, however it does not account for variations in As uptake and metabolism between individuals. Nevertheless, total urine As is still considered to be a reasonable biomarker for inorganic As exposure in clinical practice. In addition, the living environment questionnaire did not include environments such as gas stations, thermal power plants, incinerators, oil refineries, chemical plants, and plastic factories near home, which might influence heavy metal values. Finally, we could not exclude confounding factors including genetic variations, the use of medications, and the effect of other environmental pollutants, which could potentially induce renal dysfunction. 

## 5. Conclusions

In the present study, high blood Pb and high urine Cu may be associated with proteinuria and an eGFR of <60 mL/min/1.73 m^2^. High urine Ni, Mn, and Cd were also associated with proteinuria. Co-exposure to Cd and Cu, and Pb and Cr, may have synergistic effects on the association with proteinuria. Our findings should serve to remind health researchers and the government of the importance of environmental policies and legislative changes to improve human health. Future follow-up studies are necessary to clarify the causal relationships among heavy metals and proteinuria and CKD. 

## Figures and Tables

**Table 1:** Comparison of clinical characteristics among participants with and without proteinuria.

Characteristics	All (*n* = 2447)	Without Proteinuria (*n* = 2194)	With Proteinuria (*n* = 253)	*p*
Age (year)	55.1 ± 13.2	54.6 ± 13.0	59.7 ± 14.0	<0.001
Male gender (%)	39.9	39.0	48.2	0.004
DM (%)	10.5	8.4	28.5	<0.001
Hypertension (%)	25.3	23.0	45.5	<0.001
BMI (kg/m^2^)	25.0 ± 4.0	24.8 ± 3.9	26.2 ± 4.6	<0.001
SBP (mmHg)	132.1 ± 19.8	131.1 ± 19.2	140.0 ± 22.4	<0.001
DBP (mmHg)	77.5 ± 11.7	77.2 ± 11.4	80.1 ± 13.6	0.001
Occupation (%)				0.041
Agriculture, forestry, fishing, and animal husbandr	4.9	4.9	5.1	
Commerce	20.8	21.1	18.4	
Industry	11.3	11.9	6.1	
Government employees	23.0	22.9	23.5	
Service industry	6.2	6.3	4.6	
None	33.8	32.8	42.3	
Living environment (%)				
Oil-painted in the past six months	6.3	6.2	7.2	0.556
House decoration in the past six months	4.1	4.4	1.4	0.041
Burned incense	74.2	73.9	76.9	0.350
Laboratory parameters				
Fasting glucose (mg/dL)	99.9 ± 27.4	97.6 ± 23.8	119.7 ± 43.4	<0.001
Triglyceride (mg/dL)	105.0 (73.0–150.0)	102.0 (72.0–146.0)	129.0 (90.0–192.5)	<0.001
Total cholesterol (mg/dL)	199.6 ± 37.4	200.2 ± 37.2	194.9 ± 39.4	0.043
HDL-cholesterol (mg/dL)	53.0 ± 13.6	53.3 ± 13.6	49.9 ± 13.9	<0.001
LDL-cholesterol (mg/dL)	119.2 ± 34.0	119.8 ± 34.0	113.5 ± 34.0	0.005
Hemoglobin (g/dL)	14.0 ± 1.6	14.0 ± 1.6	14.0 ± 1.9	0.887
eGFR (mL/min/1.73 m^2^)	89.1 ± 16.3	90.2 ± 15.0	79.3 ± 22.8	<0.001
Uric acid (mg/dL)	5.7 ± 1.6	5.7 ± 1.5	6.2 ± 1.7	<0.001
Heavy metals				
Blood				
Pb (μg/dL)	1.6 (1.0–2.2)	1.5 (1.0–2.2)	1.9 (1.3–2.7)	<0.001
Urine				
Ni (μg/L)	2.4 (1.5–3.7)	2.3 (1.5–3.5)	3.3 (2.3–5.5)	<0.001
Cr (μg/L)	0.1 (0.1–0.1)	0.1 (0.1–0.1)	0.1 (0.1–0.1)	0.134
Mn (μg/L)	1.7 (0.9–3.0)	1.7 (0.9–2.9)	2.2 (1.2–3.5)	<0.001
As (μg/L)	78.9 (45.6–142.0)	78.2 (45.3–142.5)	82.7 (48.6–138.9)	0.760
Cu (μg/dL)	1.5 (1.0–2.0)	1.4 (1.0–1.8)	2.4 (1.9–3.5)	<0.001
Cd (μg/L)	0.8 (0.5–1.4)	0.8 (0.3–1.3)	1.1 (0.6–2.0)	<0.001

Abbreviations: DM, diabetes mellitus; BMI, body mass index; SBP, systolic blood pressure; DBP, diastolic blood pressure; HDL, high-density lipoprotein; LDL, low-density lipoprotein; eGFR, estimated glomerular filtration rate; Pb, lead; Ni, nickel; Cr, chromium; Mn, manganese; As, arsenic; Cu, copper; Cd, cadmium.

**Table 2:** Association of heavy metals with proteinuria using multivariable logistic regression analysis.

Heavy Metals	Multivariable	
	OR (95% CI)	*p*
Blood		
Pb (log per 1 μg/dL)	3.089 (1.630–5.853)	0.001
Urine		
Ni (log per 1 μg/L)	3.642 (2.285–5.807)	<0.001
Cr (log per 1 μg/L)	1.810 (0.793–4.128)	0.159
Mn (log per 1 μg/L)	2.443 (1.649–3.619)	<0.001
As (log per 1 μg/L)	0.765 (0.473–1.239)	0.277
Cu (log per 0.1 μg/dL)	1.945 (1.750–2.162)	<0.001
Cd (log per 1 μg/L)	2.671 (1.733–4.118)	<0.001

Values expressed as odds ratios (OR) and 95% confidence intervals (CI). Covariates in the multivariable model included age, sex, DM, hypertension, BMI, SBP, DBP, occupation, house decoration in the past six months, fasting glucose, log triglyceride, total cholesterol, HDL-cholesterol, LDL-cholesterol, eGFR, and uric acid (significant variables in Table 1).

**Table 3:** Comparison of clinical characteristics among participants with and without an eGFR of <60 mL/min/1.73 m^2.^

Characteristics	eGFR ≥ 60 (*n* = 2292)	eGFR < 60 (*n* = 155)	*p*
Heavy metals			
Blood			
Pb (μg/dL)	1.5 (1.0–2.2)	1.8 (1.2–2.5)	0.002
Urine			
Ni (μg/L)	2.4 (1.5–3.7)	2.7 (1.8–4.3)	<0.001
Cr (μg/L)	0.1 (0.1–0.1)	0.1 (0.1–0.1)	0.371
Mn (μg/L)	1.7 (0.9–3.0)	1.6 (0.7–2.7)	0.249
As (μg/L)	76.3 (44.1–139.0)	107.4 (73.0–177.1)	<0.001
Cu (μg/dL)	1.4 (1.0–1.9)	1.7 (1.3–2.4)	<0.001
Cd (μg/L)	0.8 (0.5–1.4)	0.8 (0.5–1.5)	0.352

Abbreviations are the same as in Table 1.

**Table 4:** Association of heavy metals with an eGFR of <60 mL/min/1.73 m^2^ using multivariable logistic regression analysis.

Heavy Metals	Multivariable	
	OR (95% CI)	*p*
Blood		
Pb (log per 1 μg/dL)	3.727 (1.207–11.510)	0.022
Urine		
Ni (log per 1 μg/L)	1.315 (0.779–2.220)	0.305
Cr (log per 1 μg/L)	0.653 (0.128–3.329)	0.608
Mn (log per 1 μg/L)	0.894 (0.539–1.482)	0.663
As (log per 1 μg/L)	0.775 (0.369–1.629)	0.502
Cu (log per 0.1 μg/dL)	1.163 (1.038–1.303)	0.009
Cd (log per 1 μg/L)	0.758 (0.404–1.423)	0.389

Values expressed as odds ratios (OR) and 95% confidence intervals (CI). Covariates in the multivariable model included age, sex, DM, hypertension, SBP, occupation, house decoration in the past six months, burned incense, fasting glucose, log triglyceride, hemoglobin, and uric acid (significant variables in Table 3).

**Table 5:** Area under the curve (AUC), cutoff value, Youden index, and sensitivity and specificity of seven heavy metals for proteinuria.

Heavy Metals	AUC (95% Confidence Interval)	Cutoff Value	Sensitivity (%)	Specificity (%)	Youden Index
Pb	0.612 (0.575–0.648) *	0.217	61.0	56.1	0.171
Ni	0.667 (0.633–0.701) *	0.455	60.6	62.5	0.231
Cr	0.513 (0.474–0.551)	−0.850	6.8	95.7	0.025
Mn	0.583 (0.546–0.620) *	0.267	57.4	54.9	0.123
As	0.509 (0.472–0.547)	1.907	51.4	51.4	0.028
Cu	0.842 (0.816–0.867) *	0.271	76.7	76.9	0.536
Cd	0.599 (0.560–0.638) *	−0.023	57.0	56.4	0.134

* *p* < 0.05. Abbreviations are the same as in Table 1.

**Table 6:** Area under the curve (AUC), cutoff value, Youden index, and sensitivity and specificity of seven heavy metals for an eGFR of < 60 mL/min/1.73 m^2^.

Heavy Metals	AUC (95% Confidence Interval)	Cutoff Value	Sensitivity (%)	Specificity (%)	Youden Index
Pb	0.580 (0.535–0.624) *	0.217	56.9	55.0	0.119
Ni	0.559 (0.514–0.603) *	0.407	52.9	53.3	0.062
Cr	0.500 (0.453–0.547)	−0.850	4.6	95.5	0.001
Mn	0.459 (0.413–0.506)	0.217	45.1	47.1	−0.078
As	0.623 (0.582–0.665) *	1.984	60.8	60.8	0.216
Cu	0.613 (0.568–0.659) *	0.192	55.6	56.8	0.124
Cd	0.515 (0.469–0.561)	−0.071	49.7	49.8	−0.005

* *p* < 0.05. Abbreviations are the same as in Table 1.

## Supplementary Materials

The following are available online at https://www.mdpi.com/2075-4418/11/2/282/s1, Table S1: The interaction effect of Cr and Pb is associated with an increase in the risk of proteinuria occurrence, Table S2: the interaction effect of Cu and Cd is associated with an increase in the risk of proteinuria occurrence.
