# Supplementary material for: Associations among Heavy Metals and Proteinuria and Chronic Kidney Disease
Source: Diagnostics (Basel). 2021 Feb 11;11(2):282. doi: 10.3390/diagnostics11020282 (PMC7918558; doi:10.3390/diagnostics11020282)
Supplement: Supplementary file 1 [file diagnostics-11-00282-s001.pdf]

Supplement Table

Table 1. The interaction effect of Cr and Pb is associated with an increased in the risk of proteinuria occurrence

Effect	OR (95% CI)	*p* value	Log_Cr by Log_Pb, *p* value
Log_Cr at Log_Pb= 0.00	0.37 (0.08-1.77)	0.212	0.048
Log_Cr at Log_Pb= 0.10	0.48 (0.13-1.84)	0.285	
Log_Cr at Log_Pb= 0.15	0.55 (0.16-1.88)	0.343	
Log_Cr at Log_Pb= 0.20	0.63 (0.21-1.94)	0.423	
Log_Cr at Log_Pb= 0.25	0.72 (0.26-2.01)	0.535	
Log_Cr at Log_Pb= 0.30	0.83 (0.32-2.11)	0.692	
Log_Cr at Log_Pb= 0.35	0.95 (0.40-2.24)	0.901	
Log_Cr at Log_Pb= 0.40	1.08 (0.49-2.40)	0.843	
Log_Cr at Log_Pb= 0.45	1.24 (0.58-2.63)	0.575	
Log_Cr at Log_Pb= 0.50	1.42 (0.68-2.95)	0.348	
Log_Cr at Log_Pb= 0.55	1.62 (0.78-3.38)	0.196	
Log_Cr at Log_Pb= 0.60	1.86 (0.87-3.97)	0.110	
Log_Cr at Log_Pb= 0.65	2.13 (0.95-4.77)	0.067	
Log_Cr at Log_Pb= 0.70	2.43 (1.02-5.83)	0.046	
Log_Cr at Log_Pb= 0.75	2.78 (1.07-7.22)	0.035	
Log_Cr at Log_Pb= 0.80	3.19 (1.12-9.05)	0.030	
Log_Cr at Log_Pb= 0.85	3.65 (1.16-11.44)	0.027	
Log_Cr at Log_Pb= 0.90	4.17 (1.20-14.55)	0.025	
Log_Cr at Log_Pb= 0.95	4.77 (1.23-18.59)	0.024	
Log_Cr at Log_Pb= 1.00	5.46 (1.25-23.85)	0.024	
Log_Cr at Log_Pb= 1.05	6.25 (1.27-30.68)	0.024	
Log_Cr at Log_Pb= 1.10	7.15 (1.29-39.57)	0.024	

The odds ratio [OR] and 95% CI were estimated by a logistic regression model. The ODDSRATIO statement produces odds ratios in the presence of interactions, and a graphical display of the requested odds ratios is produced when ODS Graphics is enabled.

Table 2. The interaction effect of Cu and Cd is associated with an increased in the risk of proteinuria occurrence

Effect	OR (95% CI)	*p* value	Log_Cr by Log_Pb, *p* value
Log_Cu at Log_Cd= -1.8	1.59 (1.15-2.20)	0.005	0.033
Log_Cu at Log_Cd= -1.6	1.66 (1.24-2.21)	< 0.001	
Log_Cu at Log_Cd= -1.4	1.73 (1.34-2.23)	< 0.001	
Log_Cu at Log_Cd= -1.2	1.80 (1.44-2.24)	< 0.001	
Log_Cu at Log_Cd= -1.0	1.87 (1.55-2.26)	< 0.001	
Log_Cu at Log_Cd= -0.8	1.95 (1.67-2.28)	< 0.001	
Log_Cu at Log_Cd= -0.6	2.03 (1.78-2.31)	< 0.001	
Log_Cu at Log_Cd= -0.4	2.12 (1.90-2.36)	< 0.001	
Log_Cu at Log_Cd= -0.2	2.20 (2.00-2.43)	< 0.001	
Log_Cu at Log_Cd= 0.0	2.30 (2.08-2.54)	< 0.001	
Log_Cu at Log_Cd= 0.2	2.39 (2.13-2.69)	< 0.001	
Log_Cu at Log_Cd= 0.4	2.49 (2.16-2.87)	< 0.001	
Log_Cu at Log_Cd= 0.6	2.59 (2.19-3.07)	< 0.001	
Log_Cu at Log_Cd= 0.8	2.70 (2.21-3.30)	< 0.001	
Log_Cu at Log_Cd= 1.0	2.81 (2.23-3.56)	< 0.001	
Log_Cu at Log_Cd= 1.2	2.93 (2.24-3.84)	< 0.001	
Log_Cu at Log_Cd= 1.4	3.05 (2.25-4.14)	< 0.001	
Log_Cu at Log_Cd= 1.6	3.18 (2.26-4.47)	< 0.001	
Log_Cu at Log_Cd= 1.8	3.31 (2.28-4.82)	< 0.001	

The odds ratio [OR] and 95% CI were estimated by a logistic regression model.

The ODDSRATIO statement produces odds ratios in the presence of interactions, and a graphical display of the requested odds ratios is produced when ODS Graphics is enabled.
